# Pre-receptor regulation of 11-oxyandrogens differs between normal and cancerous endometrium and across endometrial cancer grades and molecular subtypes

**DOI:** 10.3389/fendo.2024.1404804

**Published:** 2024-08-14

**Authors:** Marija Gjorgoska, Lea Šturm, Tea Lanišnik Rižner

**Affiliations:** Institute of Biochemistry and Molecular Genetics, Faculty of Medicine, University of Ljubljana, Ljubljana, Slovenia

**Keywords:** endometrial cancer, 11-oxyandrogens, intracrinology, androgen receptor, LC-MS/MS profiling, *in vitro* models

## Abstract

**Background:**

Endometrial cancer (EC) is a prevalent gynecological malignancy globally, with a rising incidence trend. While classic androgens have been implicated with EC risk, the role of their 11-oxygenated metabolites is poorly understood. Here, we studied 11-oxyandrogen formation from steroid precursors in EC for the first time.

**Methods:**

We performed *in vitro* studies on a panel of four EC cell lines of varying differentiation degree and molecular subtype and a control cell line of normal endometrium to assess 11-oxyandrogen formation from steroid precursors. We also characterized the transcriptomic effects of dihydrotestosterone (DHT) and 11-keto-DHT on Ishikawa and RL95-2. Key molecular players in 11-oxyandrogen metabolism and action were explored in endometrial tumors using public transcriptomic datasets.

**Results:**

We discovered that within endometrial tumors, the formation of 11-oxyandrogens does not occur from classic androgen precursors. However, we observed distinct regulatory mechanisms at a pre-receptor level in normal endometrium compared to cancerous tissue, and between low- and high-grade tumors. Specifically, *in vitro* models of low-grade EC formed higher levels of bioactive 11-keto-testosterone from 11-oxyandrogen precursors compared to models of noncancerous endometrium and high-grade, TP53-mutated EC. Moreover, the potent androgen, DHT and its 11-keto homologue induced mild transcriptomic effects on androgen receptor (AR)-expressing EC model, Ishikawa. Finally, using public transcriptomic datasets, we found *HSD11B2* and *SRD5A2*, coding for key enzymes in steroid metabolism, to be associated with better disease-specific survival, whereas higher intra-tumoral AR expression correlated with lower recurrence in TP53-wt tumors.

**Conclusions:**

The intra-tumoral metabolism of 11-oxyandrogen precursors is characteristic for low-grade EC of non-TP53-alt molecular subtypes. Our findings support further exploration of circulating 11-oxyandrogens as prognostic biomarkers in EC.

## 1 Introduction

Endometrial cancer (EC) is the most common female gynecological pathology with a concerning increase in incidence observed globally due to demographic changes (1–3). EC is classified into two major histotypes, namely endometrioid EC, which accounts for most cases and is associated with estrogen-dependency and good clinical outcome, and non-endometrioid EC, which comprises serous, clear-cell EC, carcinosarcoma, and other rarer types (4), generally regarded as estrogen-independent and associated with worse prognosis.

Within EC types, distinct molecular subtypes are distinguished: namely POLE-altered (POLE-alt), microsatellite instability-high (MSI-high, also known as mismatch repair deficient (dMMR)), tumors with non-specific molecular profile (NSMP, also known as copy number variation (CNV)-low), and TP53-altered (TP53-alt) tumors (also known as CNV-high). The former three are mainly low-grade, well differentiated, clinically favorable endometrioid tumors, whereas the latter are primarily high-grade endometrioid and serous tumors, generally characterized by a higher recurrence tendency (4, 5).

Androgen hormones are sex steroid hormones produced by the adrenal glands and gonads with broad effects on the female pre- and post-menopausal physiology. These hormones have been both directly and indirectly associated with higher EC risk [reviewed in (6)]. Apart from classic androgens, the adrenal glands produce a unique set of androgen metabolites that share an oxygen atom at C11 position and are thus called 11-oxyandrogens. These metabolites are particularly interesting in the post-reproductive female period as their levels, contrary to classic androgens remain consistent post-menopause (7, 8). 11-oxyandrogens are poorly studied in the context of EC (9).

In our study, we utilized four EC cell lines of varying degrees of differentiation and molecular subtypes and a control cell line of noncancerous endometrium to address several key questions. First, we investigated whether 11-oxyandrogens can form in EC cell lines from classic androgen precursors. Next, we examined whether 11-oxyandrogen precursors are metabolized into bioactive 11-oxyandrogens. Additionally, we characterized the transcriptomic effects of both classic and 11-oxyandrogens on EC cancer cells. Finally, we explored the expression of essential molecular players involved in 11-oxyandrogen metabolism using publicly available transcriptomic data from the Cancer Genome Atlas (TCGA) uterine corpus endometrial carcinoma (UCEC) cohort (5).

## 2 Materials and methods

### 2.1 Cell lines

The following EC model cell lines were used in this study: Ishikawa (RRID: CVCL_2529; #ECACC 99040201, Sigma Aldrich GmbH), HEC1A (RRID: CVCL_0293; #ATCC_HTB-112TM, American Type Culture Collection), RL95-2 (RRID: CVCL_0505; #CRL-1671, American Type Culture Collection), and KLE (RRID: CVCL_1329; #CRL-162, American Type Culture Collection). Ishikawa cells were derived from well differentiated, primary endometrial adenocarcinoma of a 39-year-old patient (10), HEC1A cells from moderately-well differentiated, stage IA, grade II endometrial adenocarcinoma of a 71-year-old patient (11), RL95-2 cells from moderately differentiated, grade II primary endometrial adenosquamous adenocarcinoma of a 65-year-old patient (12), and KLE cells from poorly differentiated, grade III endometrial adenocarcinoma of a 68-year-old patient (13). As a control cell line, HIEEC cells (gift from Dr. Fortier, Laval University, Canada) were used, originally established from a non-neoplastic endometrial biopsy of a 37-year-old woman (14).

The cell lines were cultured in appropriate culture media in a 37°C incubator with 5% CO_2_: HIEEC: RPMI-1640 medium (#R5886, Sigma Aldrich GmbH) supplemented with 2 mM L-glutamine (#G7513, Sigma Aldrich GmbH) and 10% fetal bovine serum (FBS, #F7524, Sigma Aldrich GmbH); Ishikawa: Eagle’s Minimum Essential Medium (#M5650, Gibco) with 5% FBS; HEC1A: McCoy’s 5A medium (#M4892, Sigma Aldrich GmbH); RL95-2: DMEM/F12 (#D6421, Sigma Aldrich GmbH) with 10% FBS, 2.5 mM L-glutamine, and 5 µg/mL insulin (#I9278, Sigma Aldrich GmbH); KLE: DMEM/F12 supplemented with 10% FBS and 2.5 mM L-glutamine, following good laboratory practices (15). All cell lines were authenticated by short tandem repeat profiling by the ATCC and routinely tested and confirmed negative for Mycoplasma contamination using the Lonza Mycoalert Mycoplasma Detection Kit. Three independent experiments were performed in cell passages less than 15.

### 2.2 Quantitative gene expression

RNA extraction from EC cell lines (from three independent experiments) was carried out using a Macherey-Nagel kit (#740933.5, Macherey-Nagel GmbH&Co, Germany), reverse-transcription with SuperScript^®^VILO™ cDNA Synthesis kit (#11754050, Invitrogen, USA). Quantitative gene expression analysis was performed using Taqman chemistry (*PAPSS2* (#Hs00989928_m1), *CYP11B1* (#Hs01596406_m1), *HSD11B2* (#Hs00388669_m1), *HSD11B1* (#Hs01547870_m1), *H6PD* (#Hs00188728_m1), all from Thermo Fisher Scientific, USA), and SYBR Green chemistry (*AR-A* (forward primer: CCAGGGAAACGAATGCAGAG; reverse primer: AGTCTCCAAACTGTGAAGCC; Sigma Aldrich), *AR-B* (forward primer: TCATCACAGCCTGTTGAACT; reverse primer: ACTGCACTTCCATCCTTGAG; Sigma Aldrich), *GPRC6A* (forward: CCGGGACATATCATAATTGGAGG; reverse: CATTGCCACTGTGACTTCTGT, Integrated DNA Technologies, USA). Expression data for other genes relevant to this study were extracted from our previously published data (16–18). Gene expression was compared using one-way ANOVA with Tukey’s HSD *post hoc* test.

### 2.3 Metabolism study in EC cell lines

HIEEC, Ishikawa, HEC1A, RL95-2, and KLE cells were seeded in 6-well plates in complete culture media. After 24h, the cells were washed with DPBS (#D8537, Sigma Aldrich), and the medium was replaced with phenol red-free, FBS-free medium. Cells were incubated with 1.6 µM dehydroepiandrosterone sulphate (DHEAS, #D5297, Sigma Aldrich GmbH), 15 nM dehydroepiandrosterone (DHEA, #A8500-00, Sigma Aldrich GmbH), 3 nM androstenedione (A4, #A6030-000, Steraloids), 15 nM 11β-hydroxy-androstenedione (11βOHA4, #A-3009, Sigma Aldrich GmbH), or 3 nM 11-keto-androstenedione (11KA4, #284998, Sigma Aldrich GmbH), for 4, 8, 24, 48, and 72 h. All steroid precursors were prepared in ethanol (#1.11727, Supelco). After each time point, the culture media were collected and stored at -80°C until further analysis. Three independent experiments were performed, each in technical duplicates. One-way ANOVA with Tukey’s HSD *post hoc* test or Kruskal-Wallis with Dunn’s test with Bonferroni correction were used where appropriate.

#### 2.3.1 Sample preparation for *in vitro* metabolism study by (liquid chromatography-tandem mass spectrometry)

Sample preparation involved liquid-liquid extraction with MTBE (#1634-04-4, Sigma Aldrich GmbH). Briefly, 1 mL of culture media was thawed and mixed with an internal standard, [^13^C_3_]-T (#730610, Sigma Aldrich GmbH). Next, 750 µL of MTBE/sample was added, and the samples were shaken for 10 min in an Eppendorf thermomixer (#5382000031, Eppendorf). After phase separation, the organic layer was collected in a separate tube; this was repeated thrice, after which samples were dried under vacuum at 45°C. Prior to LC-MS/MS analysis, samples were reconstituted in 70 µL of 70% methanol (#34966, Honeywell/Riedel-de Haen) in water (#1.15333, Supelco) with 0.2 mM NH_4_F (#52481, Honeywell/Fluka).

For DHEAS, we performed solid-phase extraction (SPE) using 100 µL of culture media, to which DHEAS-d_5_ (#D-066, Cerilliant) was added as an internal standard. SPE included: column conditioning (#8B-S001-EAK, Phenomenex) with 1 mL of methanol, equilibration with 1 mL of water, sample loading, column drying for 10 min, and elution with 1.5 mL methanol. Subsequently, samples were evaporated under vacuum at 45°C and reconstituted in 150 µL of 70% methanol with 0.2 mM NH_4_F before LC-MS/MS.

#### 2.3.2 LC-MS/MS metabolic profiling

Two LC-MS/MS methods were developed for *in vitro* metabolic profiling, one in positive electrospray ionization mode (ESI) for DHEA, A4, testosterone (T, #B6500, Sigma Aldrich GmbH), 5α-dihydrotestosterone (DHT, #A2579-000, Steraloids), 11βOHA4, 11KA4, 11β-hydroxy-testosterone (11βOHT, #A5760-000, Steraloids), 11-keto-testosterone (11KT, #K8250, Sigma Aldrich GmbH), and 11-keto-dihydrotestosterone (11KDHT, #A2375-000, Steraloids), the second method was for DHEAS analysis in ESI-negative mode. Compound- and instrument-specific parameters are given in **Supplementary Tables 1A, B**. The LC-MS/MS methods were not validated as per guidelines for analytical method validation.

Chromatographic separation was performed on a Shimadzu Nexera XR HPLC system (Shimadzu Corporation, Kyoto, Japan) with a Kinetex 2.6 µm XB-C18 (100 × 4.6 mm) column (#00D-4496-E0, Phenomenex). Mobile phase A (5% methanol in H_2_O, 0.2 mM NH_4_F) and B (methanol, 0.2 mM NH_4_F) were used in both methods, but with a different gradient elution profile (see **Supplementary Tables 1A, B**). The column temperature was set to 45°C for the ESI-positive mode method and 38°C for DHEAS. In both methods, the total solvent flow was set at 0.5 mL/min, the injection volume was 25 µL.

The MS analysis was performed on a Sciex 3500 Triple Quadrupole system (AB Sciex Deutchland GmbH, Darmstadt, Germany). The LLOQ for each analyte was defined as the lowest calibration point with accuracy ± 20% of nominal concentration and is given in **Supplementary Tables 1A, B**. Data acquisition and analysis were performed using the Analyst 1.6 software. Calibrators ranging from 5 pg/mL to 250 ng/mL (or in the case of DHEAS, 5 pg/mL to 500 ng/mL) were prepared in cell culture media and extracted as samples. 1/x weighing, and linear least squares regression was used to produce standard curves.

### 2.4 RNA-sequencing

Ishikawa and RL95-2 cells were cultured in complete media for 24 hours, followed by incubation with 10 nM DHT, 10 nM 11KDHT or ethanol (as control) for 48 hours. RNA extraction was carried out using a Macherey Nagel kit, following the manufacturer’s instructions. mRNA sequencing was performed on an Illumina platform at Novogene Inc. Non-directional poly-A library preparation was used. Quality control of raw reads and read mapping to the reference genome were conducted using fastp and Hisat2 v2.0.5 software, respectively. Gene counts were obtained using featureCounts v1.5.0-p3. Differential gene expression analysis was performed using the DeSeq2 package in R studio (19). Differentially expressed genes were identified based on a fold-change threshold greater than 1.5 (absolute value) and an adjusted p-value (with Benjamini-Hochberg (BH) method) of less than 0.01. Three independent experiments were performed.

### 2.5 TCGA uterine corpus endometrial carcinoma dataset

The open-access TCGA database of primary endometrial tumors from Kandoth et al. (5), was accessed through the University of California San Francisco Xena browser. Differential gene expression analysis of raw counts of protein-coding genes was performed using the DeSeq2 package in R studio (19). Differentially expressed genes were identified based on a fold-change threshold greater than 2 (absolute value) and an adjusted p-value (BH method) of less than 0.01. Pathway activity scores were inferred on log transformed, Fragments Per Kilobase Milion FPKM-upper quartile normalized (FPKM-uq) values using single-sample gene set enrichment analysis (ssGSEA) implemented in the GSVA R package (20, 21), and hallmark gene sets from the Molecular Signatures Database (MSigDB) (22). Differences in pathway activity scores between groups were analyzed using the Limma R package by moderated t-test with BH correction for multiple testing (23); adjusted p values less than 0.01 were considered significant.

Optimal cutoff points of RNA expression levels in relation to survival were determined with the maxstat package in R studio (24). Survival plots were generated using the Kaplan-Meier method. Uni-and multivariate Cox proportional hazards models were fitted to estimate hazard ratios. P-values were two-sided, confidence intervals were calculated at the 95% level, and significance was defined as <0.05.

### 2.6 Single cell RNA-seq dataset of endometrioid EC

Single-cell RNA-seq data from (25) were downloaded from GEO (GSE173682) and involved five endometrioid tumors. The analysis was performed using the Seurat R package (26), and involved filtering of cells with unique feature counts >2,500 and <200, and cells with >25% mitochondrial counts. The filtered count matrices were then normalized and scaled. The top 2,000 most variable genes were summarized by PCA into 50 principal components (PCs). To identify cell clusters, graph-based Louvain clustering was performed with all 50 PCs, and Seurat’s FindClusters function with a resolution of 0.7.

### 2.7 Statistical analysis

Statistical analysis and visualization were performed using R studio version 4.3.0 or higher. The statistical methods are described in the methods section and figure legends. All p values were two-sided.

## 3 Results

### 3.1 Classic androgen precursors cannot be metabolized to 11-oxyandrogens intra-tumorally

**Figure 1:**
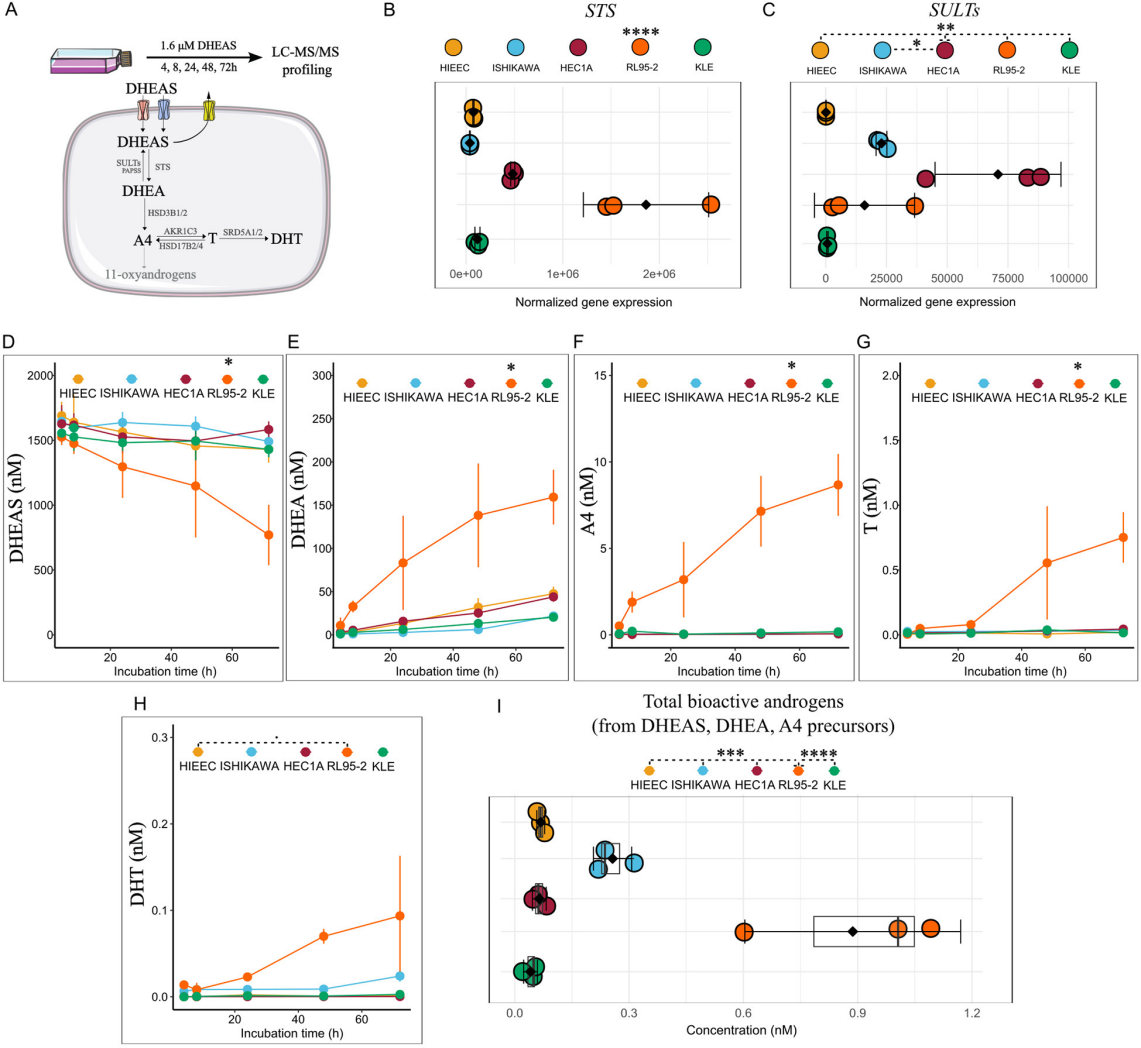
DHEAS utilization potential by EC cell lines and control HIEEC. **(A)** Workflow of the metabolism study. **(B, C)** Normalized gene expression of enzymes in the first step of DHEAS metabolism (SULTs as sum of SULT2A1 and SULT2B1), n=3, extracted from [26, 28]. **(D–H)** Line plots showing formed metabolites upon incubation of control HIEEC and EC cell lines with 1.6 µM DHEAS over time (n=3, each in technical duplicate). **(I)** Sum of bioactive androgens, T and DHT formed from separate incubation with classic androgen precursors, DHEAS (1.6 µM), DHEA (15 nM) and A4 (3 nM) in control and EC cell lines (n=3, each in technical duplicate). Data are represented as mean ± SD **(B–I)** and raw data as dots in **(B, C)**. *p<0.05, **p<0.01, ***p<0.001; ****p<0.0001 by One-Way ANOVA with Tukey’s Honestly Significant Difference (HSD) *post-hoc* test (B-I; statistical analysis in **(D–G)** represented for the final incubation time point, 72h). A4, androstenedione; DHEA, dehydroepiandrosterone; DHEAS, DHEA-sulfate; DHT, 5α-dihydrotestosterone; T, testosterone.

We investigated whether classic androgen precursors, including DHEAS, DHEA and A4 can be metabolized to 11-oxyandrogens intra-tumorally using a panel of EC model cell lines representing low-grade, POLE-alt EC - Ishikawa, low-grade, MSI-high EC – HEC1A and RL95-2, high-grade, TP53-alt EC - KLE, and a control cell line HIEEC. For this purpose, we first examined the expression of enzymes involved in the conversion of classic androgen precursors to bioactive classic and 11-oxyandrogens (**Figures 1B, C**, **Supplementary Figure 2A**). Importantly, *CYP11B1*, coding for the enzyme that catalyzes 11β-hydroxylation of classic androgens (A4 and T) in the adrenal cortex was not expressed in any of the cell lines (**Supplementary Figure 2A**), indicating that intra-tumoral 11-oxyandrogen formation is not feasible.

Next, we incubated the cell lines with physiologically relevant concentrations of classic androgen precursors: 1.6 µM DHEAS, 15 nM DHEA, and 3 nM A4 over a 72-hour period (**Figure 1A**, **Supplementary Figure 1D**), followed by LC-MS/MS profiling of formed metabolites. Indeed, we confirmed the absence of 11-oxyandrogen formation from DHEAS, DHEA and A4 by LC-MS/MS. Moreover, we observed that EC cell lines had different potential to metabolize classic precursors to bioactive androgens, which was not related to tumor grade or molecular phenotype.

In terms of DHEAS metabolism, we observed RL95-2 cells to metabolize a higher percentage of this precursor to downstream metabolites compared to the rest of cell lines (**Figure 1D**). This could be explained by significantly higher *STS* expression in this cell line (**Figure 1B**). Consequently, the levels of DHEA, the first downstream metabolite of DHEAS, as well as those of A4, T and DHT were highest in RL95-2 compared to the control cell line, HIEEC, and the cancer cell lines, Ishikawa, HEC1A and KLE (**Figures 1E–H**). Here, it should be noted that the levels of DHEA, A4 and those of the bioactive androgens T and DHT that formed from 1.6 µM DHEAS in RL95-2 in 72 hours were relatively low (DHEA ≈ 150 nM, A4 <10 nM, T <1 nM, DHT <0.1 nM), and did not account for the whole DHEAS that was metabolized. This might be explained by the low levels of *HSD3B1/2* (**Supplementary Figure 2A**) and high expression of *AKR1C3*, leading to DHEA being shunted towards 5-androstenediol (5-Adiol), which was not profiled in our assay. Of note, the catalytic efficiency was reported to be only two-fold lower for DHEA conversion to 5-Adiol (k_cat_/Km: 12 ± 1.9 min^-1^ µM^-1^) as compared with A4 conversion to T (k_cat_/Km: 23 ± 3.1 min^-1^ µM^-1^) (27).

Moreover, DHEA can be hydroxylated at C16 position by CYP3A4/7 (28) and also 16α-DHEA was not measured in our assay. In addition, conjugation of metabolites formed from DHEAS, primarily glucuronidation, mediated by UGT2B isoforms 7, 15 and 17 (29), can be also involved and account for the rest of DHEAS metabolites. However, the expression of *CYP3A4*, *CYP3A7*, *UGT2B7*, *UGT2B15* and *UGT2B17* in RL95-2, and the other EC cell lines included in our study, is very low, as seen in the Cancer Cell Line Encyclopedia (CCLE) data [(30), https://depmap.org/portal/]. Finally, the cancer cell lines Ishikawa, HEC1A and KLE and the control cell line, HIEEC, metabolized DHEAS to low amounts of DHEA whereas A4, T or DHT practically did not form (**Figures 1E–H**).

In terms of DHEA metabolism, we observed RL95-2 cells to metabolize a higher percentage of this precursor to downstream metabolites compared to other cell lines (**Supplementary Figure 1A**). In this cell line, less than 10% DHEA proceeded to A4, and subsequently to low levels of T (**Supplementary Figure 1B**). This can be explained by expression of *HSD3B1/HSD3B2* and *AKR1C3* (**Supplementary Figure 2A**). Apart from RL95-2, HEC1A cell line also expressed comparable levels of *HSD3B2* to RL95-2, however, did not form A4 from DHEA. This could be due to higher expression of *AKR1C3* in this cell line, which converts A4 to T and further conjugation with glucuronic acid or sulfate. Alternatively, it can involve DHEA conversion to 5-Adiol via AKR1C3 (27).

The formation of bioactive T from A4, on the other hand, was highest in low-grade, *AKR1C3*-high cell line, Ishikawa but not in low-grade, *AKR1C3*-high model, HEC1A (**Supplementary Figures 1E, F**). This could be explained by the high *SRD5A1* levels in HEC1A, (**Supplementary Figure 2A**), which potentially shunts A4 to 5α-androstenedione (not profiled in our LC-MS/MS method) instead of T. The levels of DHT from DHEA and A4 were below detection in all cell lines. Altogether, the low-grade, MSI-high cell line, RL95-2 formed higher levels of bioactive androgens from classic androgen precursors compared to the rest (**Figure 1I**).

### 3.2 11-oxyandrogen precursors are metabolized *in-situ* in low-grade *in vitro* models but not in noncancerous endometrium or high-grade, TP53-alt tumor model

**Figure 2:**
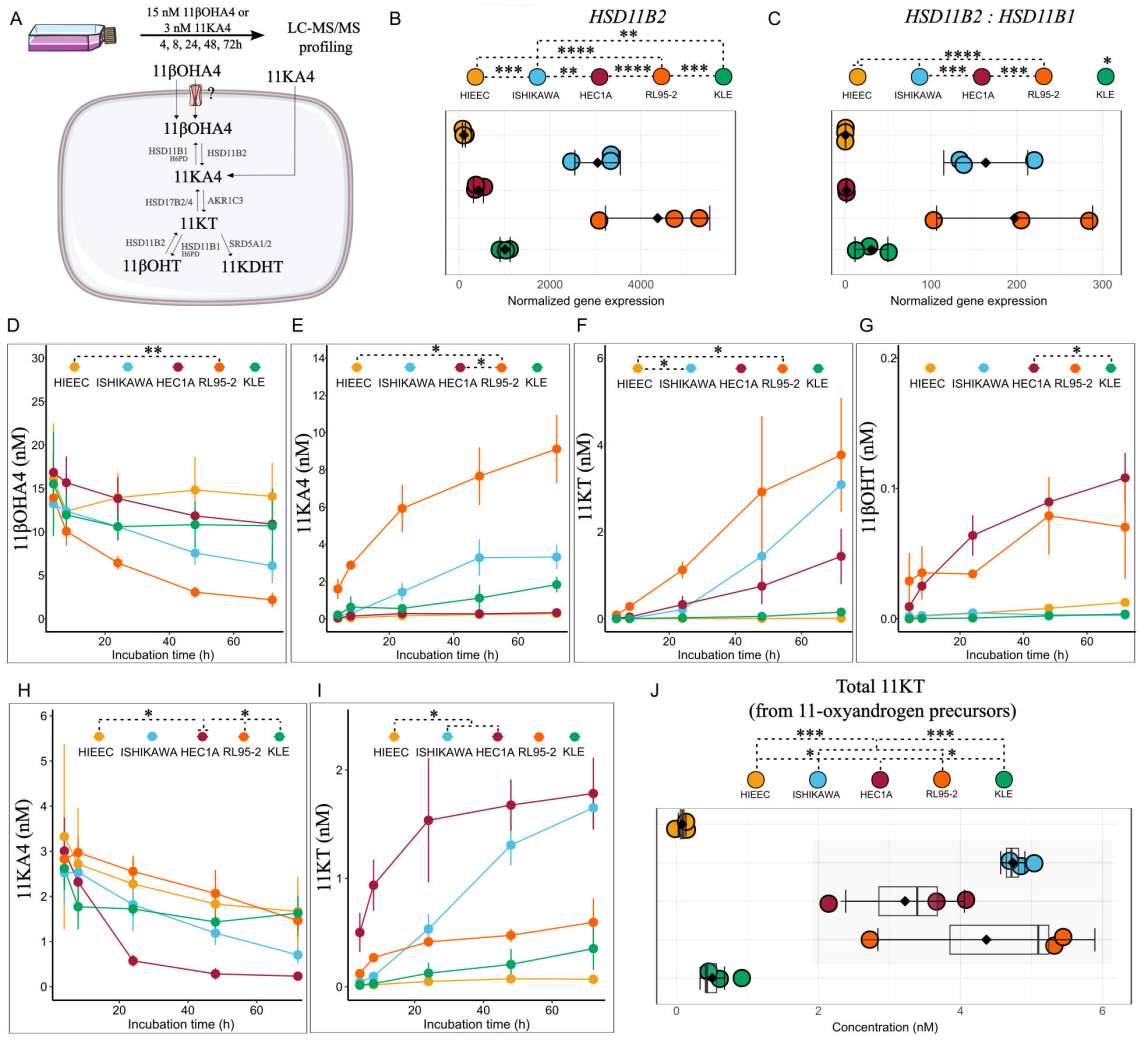
11-oxyandrogen precursor utilization potential by EC cell lines and control HIEEC cells. **(A)** Workflow of the metabolism study. **(B, C)** Normalized gene expression and ratio of enzymes in the first step of 11βOHA4 metabolism (n=3, each in technical triplicate). **(D–G)** Line plots showing metabolites formed upon incubation of EC cell lines and control HIEEC with 15 nM 11βOHA4 (n=3, each in technical duplicate). **(H, I)** Line plots showing 11KT formed upon incubation of EC cell lines and control HIEEC with 3 nM 11KA4 (n=3, each in technical duplicate). **(J)** Sum of bioactive 11-oxyandrogen, 11KT formed upon separate incubation with 11βOHA4 (15 nM) and 11KA4 (3 nM) in control and EC cell lines for 72h (n=3, each in technical duplicate). Data are the mean ± SD **(B–J)** and raw data as dots in (B-C, J). *p<0.05, **p<0.01, ***p<0.001; ****p<0.0001 by One-Way ANOVA with Tukey’s Honestly Significant Difference (HSD) *post hoc* test **(B, C, J)** and Kruskal-Wallis followed by Dunn’s *post hoc* test with Bonferroni correction (D-I, statistical analysis represented for the final incubation time point, 72h). 11βOHA4, 11β-hydroxy-androstenedione; 11βOHT, 11β-hydroxy-testosterone; 11KA4, 11-keto-androstenedione; 11KT, 11-keto-testosterone.

We next wondered whether endometrial tumors could metabolize 11-oxyandrogen precursors, including 11βOHA4 and 11KA4 to AR-activating 11-oxyandrogens, such as 11KT and 11KDHT. To explore this, we incubated the same panel of EC cell lines with physiologically relevant concentrations of 11βOHA4 (15 nM) and 11KA4 (3 nM), for a total of 72 h (**Figure 2A**).

The gene expression of key enzymes of 11-oxyandrogen metabolism differed significantly between cell lines. More specifically, *HSD11B2*, coding for the enzyme that catalyzes 11βOHA4 oxidation to 11KA4, was highest in low-grade Ishikawa and RL95-2 cells (**Figure 2B**) but not in low-grade HEC1A cells. In contrast, the control cell line HIEEC expressed highest levels of *HSD11B1*, which encodes the enzyme that catalyzes the reverse reaction (**Figure 2C**; **Supplementary Figure 2A**).

In accordance with *HSD11B2* expression levels RL95-2 formed highest levels of 11KA4 from 11βOHA4, followed by Ishikawa and KLE (**Figure 2E**), (**Figure 2B**). The levels of 11KT formed from 11KA4 via AKR1C3 were greatest in RL95-2 and Ishikawa, followed by HEC1A (**Figure 2F**). *AKR1C3* levels were highest in Ishikawa and HECA1, followed by RL95-2 (**Supplementary Figure 2**). Of note, the low levels of 11KA4 observed in HEC1A suggest fast conversion of this metabolite to 11KT, which could be explained by high *AKR1C3* expression in this cell line (**Supplementary Figure 2A**). Low levels of 11βOHT were detected in HEC1A and RL95-2 (**Figure 2G**). Notably, the high-grade, TP53-alt cell line KLE metabolized 11βOHA4 only to 11KA4, whereas the control cell line HIEEC practically did not metabolize the precursor at all, due to very low *HSD11B2* levels (**Figures 2D–G**).

We also incubated cells with 3 nM 11KA4, which has similar levels to A4 in the systemic circulation. *AKR1C3*-high cell lines Ishikawa and HEC1A formed highest levels of 11KT from 11KA4 (**Figures 2H, I**). RL95-2 formed low 11KT levels from 11KA4, probably due to due to high expression of *HSD17B2*, which catalyzes 11KT conversion back to 11KA4 (31) (**Supplementary Figure 2A**). Of note, 11KDHT levels were below the detection limit upon incubation with 15 nM 11βOHA4 or 3 nM 11KA4.

Altogether, cell lines of low-grade, non-TP53-alt EC formed higher 11KT levels from 11-oxyandrogen precursors than the high-grade, TP53-alt cell line, and the control cell line (**Figure 2J**). High *HSD11B2* and *AKR1C3* expression conferred high metabolizing potential of 11-oxyandrogen precursors. Notably, the amount of 11KT formed from 11-oxyandrogen precursors was several folds higher than T formed from classic androgen precursors. This highlights intra-tumoral 11-oxyandrogen metabolism as an important source of AR-activating hormones in endometrial tumors.

### 3.3 Classic and 11-oxygenated bioactive androgens induce mild transcriptomic effects on *in vitro* EC models

**Figure 3:**
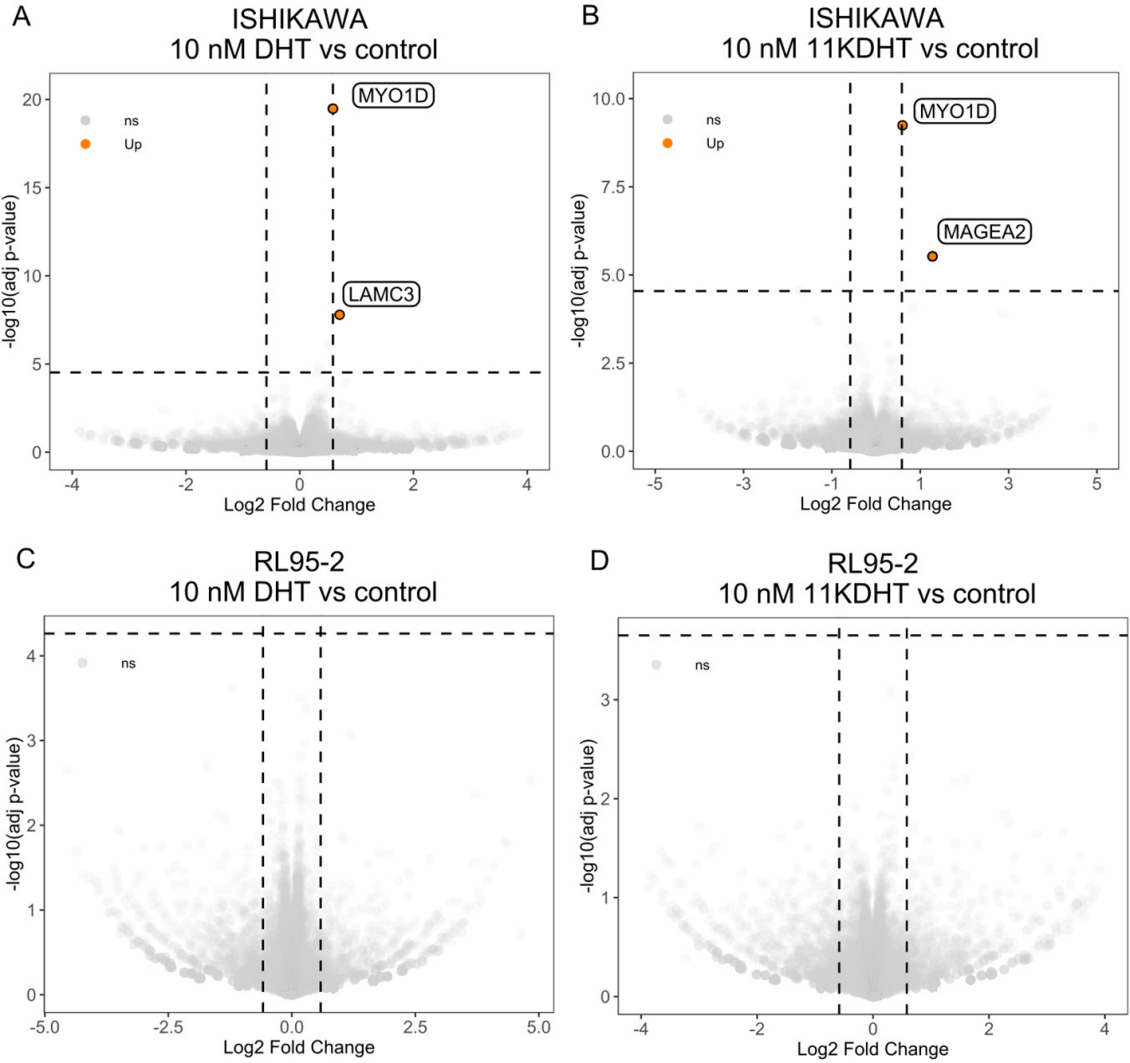
Transcriptomic changes induced by classic and 11-oxyandrogens on *in vitro* models of EC. **(A)** Differentially expressed genes upon incubation of Ishikawa cells with 10 nM DHT. **(B)** Differentially expressed genes upon incubation of Ishikawa cells with 10 nM 11KDHT. **(C)** Differentially expressed genes upon incubation of RL95-2 cells with 10 nM DHT. **(D)** Differentially expressed genes upon incubation of RL95-2 cells with 10 nM 11KDHT. The horizontal dashed line indicates the false discovery threshold (BH adjusted p value <0.01); the vertical dashed lines indicate fold-change threshold (greater than 1.5 (absolute value)). *LAM3C*, laminin subunit gamma 3; *MYO1D*, myosin ID; *MAGEA2*, melanoma-associated antigen 2.

To investigate the transcriptomic effect of androgen and 11-oxyandrogen signaling on endometrial cancer cells, we performed mRNA sequencing upon 48h incubation with 10 nM DHT, 10 nM 11KDHT or ethanol as control of two model cell lines, namely Ishikawa, which expressed *AR* (**Supplementary Figure 2A**) and RL95-2, which was the most efficient in metabolizing androgen and 11-oxyandrogen precursors and expressed low *AR* levels (**Figures 1**, **2**). In both cell lines, the transcriptomic effects of DHT and 11KDHT were mild, without significantly altered signaling pathways. The lists of differentially expressed genes are given in **Supplementary Tables 2A–D**. No significantly expressed genes were observed in RL95-2 cells upon incubation with either DHT or 11KDHT (**Figures 3C, D**)

In the *AR*-expressing, low-grade EC model, Ishikawa, both DHT and 11KDHT induced upregulation of *MYO1D*, coding for unconventional myosin ID protein (**Figures 3A, B**). Furthermore, incubation with 10 nM DHT in Ishikawa cells also caused upregulation of *LAMC3*, coding for laminin subunit gamma 3 (**Figure 3A**). Incubation with 10 nM 11KDHT, apart from *MYO1D* upregulation, also induced changes in *MAGEA2* gene, coding for melanoma-associated antigen 2 in Ishikawa (**Figure 3B**).

### 3.4 Low-grade endometrial tumors have heightened potential of metabolizing 11-oxyandrogen precursors compared to tumor-adjacent endometrium

We next assessed the expression of key enzymes of the 11-oxyandrogen metabolism in primary endometrial tumors and tumor-adjacent tissues from the TCGA UCEC cohort (5). A list of differentially expressed genes between tumor-adjacent endometrium and endometrial tumors of low-grade and high-grade EC can be found in **Supplementary Tables 3A, B**.

**Figure 4:**
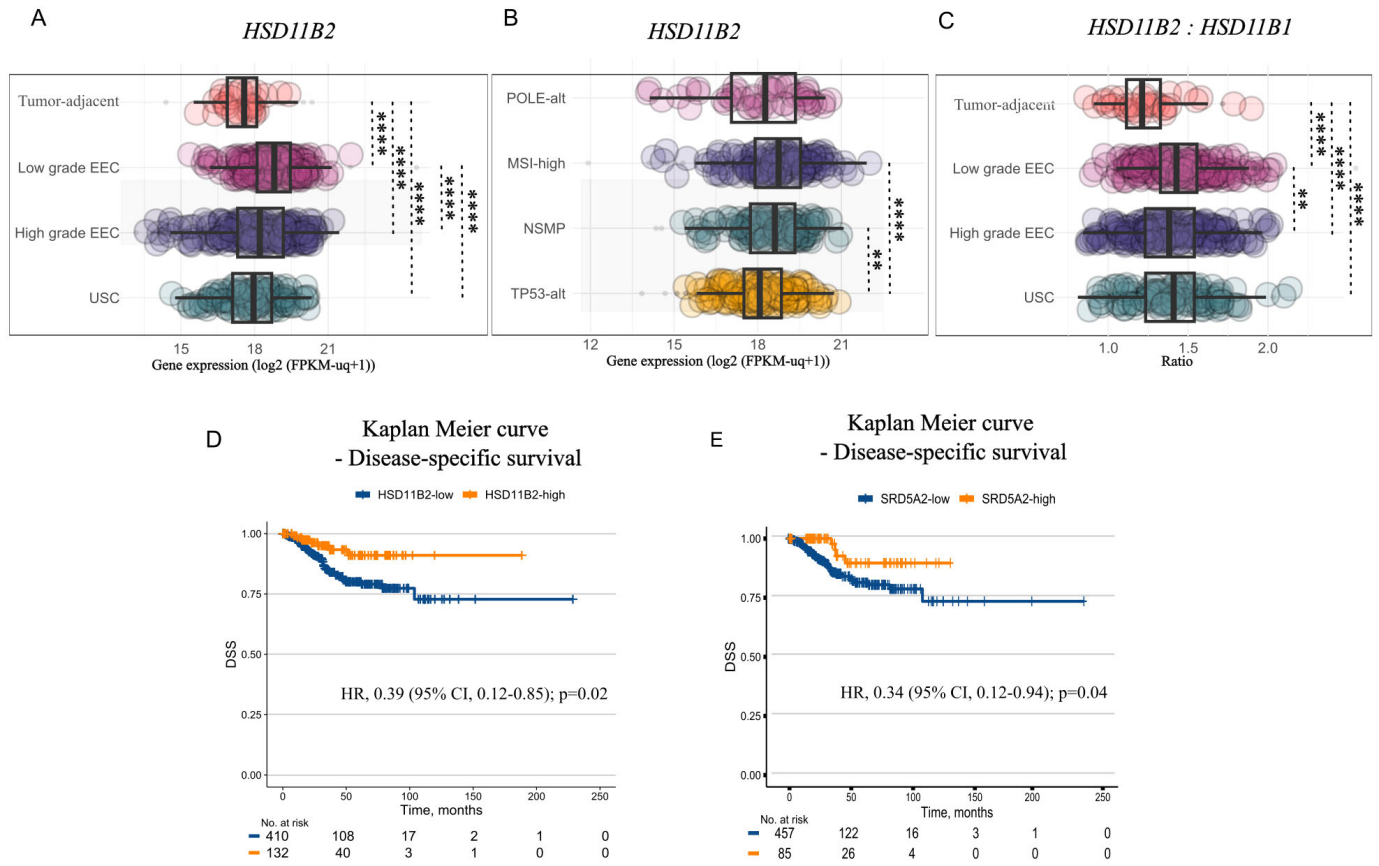
*In-situ* utilization potential of 11-oxyandrogen precursors. **(A)** Boxplot illustrating *HSD11B2* expression in tumor-adjacent endometrial tissue (n=35), low-grade endometrioid EC (EEC) (n=215), and high-grade EC, including high-grade EEC (n=194), and uterine serous carcinoma (USC) (n=113) from the TCGA UCEC cohort. **(B)** Boxplot illustrating *HSD11B2* expression in POLE-alt (n=48), MSI-high (n=142), NSMP (n=141), and TP53-alt (n=151) tumors from the TCGA UCEC cohort. **(C)** *HSD11B2: HSD11B1* in tumor-adjacent and cancerous endometrial tissue of different grades (n=same as in A) from the TCGA UCEC cohort. **(D)** Kaplan-Meier plot showing disease-specific survival (DSS) of patients with *HSD11B2*-high (n=132) vs HSD11B2-low tumors (n=410) from the TCGA UCEC cohort. **(E)** DSS in patients with SRD5A2-high (n=85) vs SRD5A2-low tumors (n=459) for the TCGA UCEC cohort. Gene expression is expressed in log2(FPKM-uq+1). Data is represented as boxplots showing median, 1st and 3rd quartiles, whiskers as min-max values and raw data as individual dots in **(A–C)**. Significance levels: **p<0.01, ****p<0.0001 by Kruskal-Wallis followed by Dunn’s *post hoc* test with Bonferroni correction for **(A–C)**, univariate Cox proportional hazard model adjusted for grade in **(D, E)**.

We found that endometrial tumors of both low- and high-grade have significantly higher *HSD11B2* levels than tumor-adjacent endometrium (**Figure 4A**), which, like the control cell line HIEEC, displayed low *HSD11B2/HSD11B1* ratio compared to EC of any grade (**Figure 4C**). Furthermore, *HSD11B2* expression differed between grades and molecular subtypes, being most pronounced in low-grade endometrioid tumors (**Figure 4A**) and in MSI-high and NSMP molecular subtypes (**Figure 4B**). Importantly, *HSD11B2* expression was also associated with better disease-specific survival (DSS) (Univariate Cox proportional hazard model adjusted for grade: Hazard Ratio [HR] 0.39, 95% CI, 0.12-0.85, p=0.02) (**Figure 4D**).

The downstream utilization of circulating or locally formed 11KA4 is also dependent on the expression of relevant enzymes, including AKR1C3, which catalyzes the bioactivation of (11-oxy)-A4 to 11KT, among others. *AKR1C3* was slightly upregulated in endometrial tumors compared to tumor-adjacent tissue (**Supplementary Figures 3A, B**), suggesting greater 11KA4 metabolism potential in tumors than tumor-adjacent endometrium. Of note, not all endometrial tumors from the TCGA dataset had a matching tumor-adjacent tissue, therefore, the latter comparison may not be optimal. Finally, expression of *SRD5A2*, coding for a key enzyme that catalyzes the formation of the most potent androgen, DHT, and its 11-oxyhomologue, 11KDHT, was associated with lower pro-tumoral cellular pathway activity (**Supplementary Figures 3C, D**), and better DSS vs. patients with SRD5A2-low tumors (Univariate Cox proportional hazard model adjusted for grade: Hazard Ratio [HR] 0.34, 95% CI, 0.12-0.94, p=0.04) (**Figure 4E**). *SRD5A1*, on the other hand, remained unchanged in low-grade EC compared to tumor-adjacent endometrium. However, it was upregulated in high-grade tumors compared to tumor-adjacent endometrium; however, the change was below the set threshold of an absolute 2-fold change (Fold change: 1.6; adjusted p-value: 3.82 × 10^-8^). Altogether, the data on EC tumor tissue expression levels as well as our *in vitro* data suggest that *in situ* 11-oxyandrogen metabolism is characteristic for tumors of lower grade and clinically more favorable molecular subtypes of EC.

### 3.5 In EC, androgen receptor (AR) expression is associated with favorable disease parameters

Based on the mild transcriptomic effects that we observed upon incubation of Ishikawa and RL95-2, we suspected that (11-oxy)-androgen signaling might not primarily affect the epithelial cancer cell population. In continuation we examined AR expression and activity across EC grades and molecular subtypes using transcriptomics data from (5) and scRNA-seq data from Regner et al. (25).

**Figure 5:**
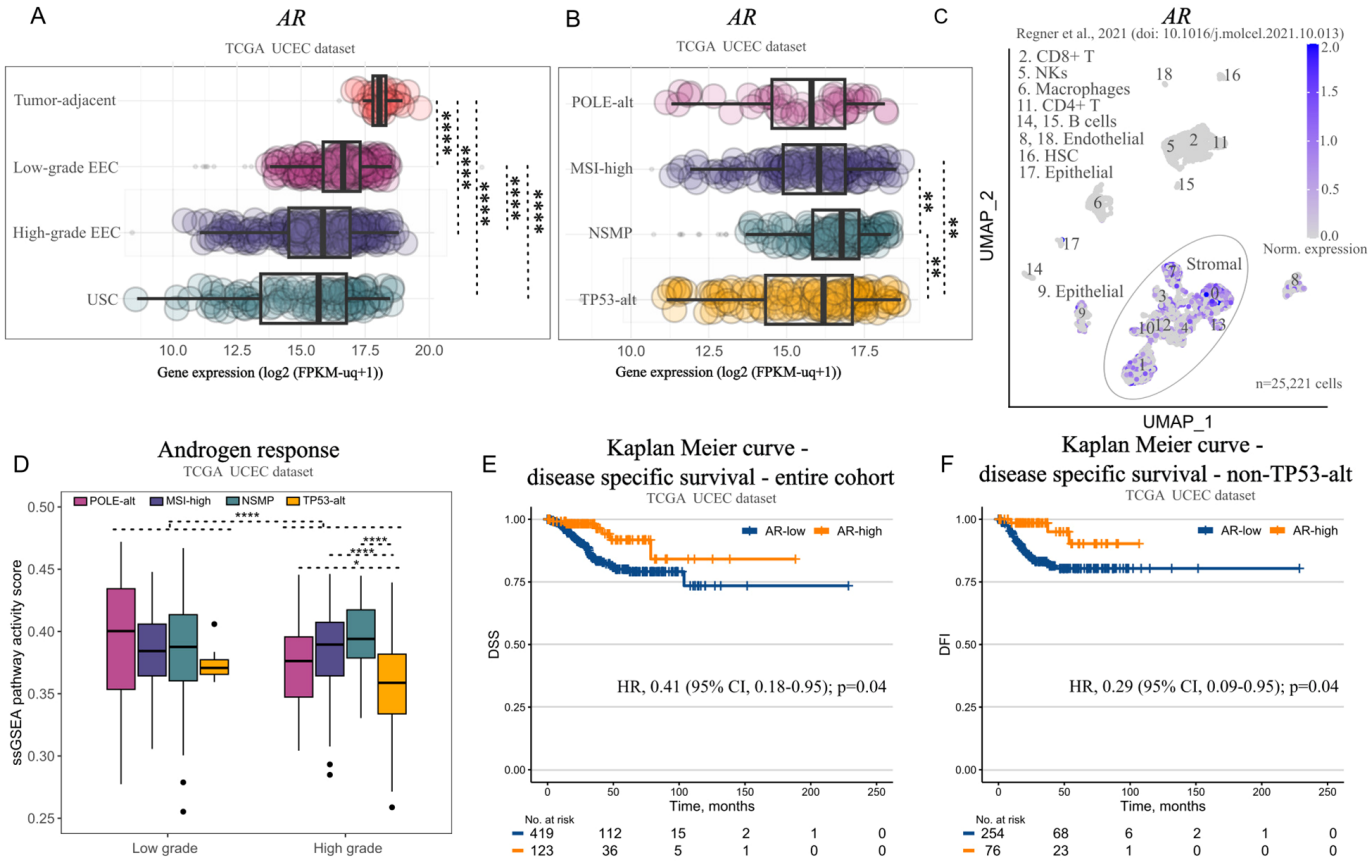
Androgen receptor (AR) signaling in endometrial tumors and its association with survival. **(A)** Boxplot illustrating AR expression in tumor-adjacent endometrial tissue (n=35), low-grade endometrioid EC (EEC) (n=215), high-grade EEC (n=194), and uterine serous carcinoma (USC) (n=113) from the TCGA UCEC cohort. **(B)** Boxplot illustrating AR expression in POLE-alt (n=48), MSI-high (n=142), NSMP (n=141), TP53-alt (n=151) tumors from the TCGA UCEC cohort. **(C)** 2D UMAP plot showing *AR* expression across different cell populations in endometrioid tumors (n=5) from Regner et al. **(D)** Boxplot showing androgen pathway activity in molecular subtypes of EC, stratified by grade (n=196 low-grade; from them: 17, POLE-alt, 63, MSI-high, 108, NSMP, 8, TP53-alt; n=286 high-grade; from them: 31, POLE-alt, 79, MSI-high, 33, NSMP, 143, TP53-alt) from the TCGA UCEC cohort. **(E)** Kaplan-Meier plot showing disease-specific survival (DSS) in patients with AR-high (n=123) vs AR-low tumors (n=419) from the TCGA UCEC cohort. **(F)** Kaplan-Meier plot showing DSS of patients with AR-high (n=76) vs AR-low non-TP53-alt tumors (n=254) from the TCGA UCEC cohort. Data is represented as boxplots showing median, 1^st^ and 3^rd^ quartiles, whiskers as min-max in **(A, B, D)** Significance levels: **p<0.01, ****p<0.0001 by Kruskal-Wallis followed by Dunn’s *post hoc* test with Bonferroni correction for A, B, D; univariate Cox proportional hazard model adjusted for grade in E-F. HSC, hematopoietic stem cells; NKs, natural killer cells.

*AR* expression was highest in tumor-adjacent endometrium and lowest in high-grade tumors, G3 EEC and USC (**Figure 5A**). In terms of molecular subtype, NSMP tumors displayed the highest *AR* expression compared to the rest (**Figure 5B**). In terms of cell populations, we found *AR* expression to be low in epithelial cells and immune cell populations, but prominent in stromal populations, which comprise a great portion of the tumor mass and might be the main target of bioactive (11-oxy)-androgens (**Figure 5C**).

Finally, we inferred the responsiveness of endometrial tumors to androgens using bulk transcriptomics data of the TCGA UCEC cohort. Unsurprisingly, low-grade tumors, which expressed highest *AR* levels were more responsive to androgens than high-grade tumors (**Figure 5D**). Moreover, within the high-grade subset, those with a TP53-alt molecular profile were less responsive to androgens compared to high-grade tumors with unaltered TP53 (**Figure 5D**). Finally, *AR* expression was associated with better DSS (Univariate Cox proportional hazard model adjusted for grade: HR 0.41, 95% CI, 0.18-0.95, p=0.04) (**Figure 5E**), whereas patients with *AR*-enriched, non-TP53-alt tumors had better disease-free interval (DFI) comparing to *AR*-low counterparts (Univariate Cox proportional hazard model adjusted for grade: HR 0.29, 95% CI, 0.09-0.95, p=0.04) (**Figure 5F**).

## 4 Discussion

In our study, we analyzed extensively the profile of metabolites formed in a panel of EC model cell lines with varying degrees of differentiation and molecular phenotype, upon incubation with physiologically relevant levels of classic androgen and 11-oxyandrogen precursors. Our findings indicate that intra-tumoral formation of 11-oxyandrogens from classic androgen precursors does not occur. However, we observed that low-grade *in vitro* models form higher levels of bioactive 11KT from 11-oxyandrogen precursors, unlike normal, noncancerous endometrium or high-grade, TP53-altered models. This provides the rationale of investigating further 11-oxyandrogens in blood or biological fluids near endometrial tumors for their prognostic potential.

Additionally, we investigated the transcriptomic changes induced by potent classic and 11-oxyandrogens on cancerous endometrial epithelial cells. In the AR-expressing, low-grade EC cell model Ishikawa, treatment with DHT and 11KDHT led to the upregulation of the *MYO1D* gene. Notably, the unconventional myosin 1D, has been implicated in promoting carcinogenesis by anchoring the epithelial growth factor receptor (EGFR) to the plasma membrane in colorectal cancer model (32). Furthermore, other members of the class I myosin family, such as MYO1B (33) and MYO1E (34), have been associated with poorer survival outcomes in colorectal cancer and lung adenocarcinoma, respectively.

Besides *MYO1D* upregulation, DHT and 11KDHT induced same-directional transcriptional changes in other genes but with varying intensities. More specifically, both upregulated *LAMC3* by 1.5- and 1.4-fold, respectively; but this change was above the false discovery rate only for DHT. Similarly, DHT and 11KDHT increased *MAGEA2* expression by 1.9- and 2.4-fold, respectively; this remained significant only for 11KDHT. The differences in the intensity of the transcriptional effects induced by DHT and 11KDHT might be due to their different affinity for AR, as was reported for T and DHT (35, 36). Additionally, these two ligands might affect AR’s specificity and affinity for androgen response elements as well as for co-regulators, leading to differential expression of androgen target genes (35, 36). Lastly, they might be metabolized differently within cells, resulting in varying intracellular concentrations and durations of activity, further contributing to the observed differences in gene expression (37).

Furthermore, by analyzing a large cohort of over 500 EC patients from TCGA, we found that higher tumoral expression of *HSD11B2* and *SRD5A2* is associated with better DSS in EC, suggesting these enzymes may have positive prognostic potential. This association warrants further investigation. The potential mechanisms through which HSD11B2 and SRD5A2 contribute to better clinical outcomes in EC patients probably involve multiple steroid hormone classes, beyond androgens, due to the interconnected nature of steroid metabolism. For instance, HSD11B2 not only converts weak 11β-hydroxy-androgens to more potent 11-keto-androgens but also regulates glucocorticoid signaling by inactivating cortisol to cortisone (28). This dual role implies that HSD11B2’s association with improved survival could be linked to both intra-tumoral androgen and glucocorticoid signaling pathways. Similarly, SRD5A2 plays a role in the formation of potent androgens, such as DHT and 11KDHT. Additionally, SRD5A2 converts progesterone to the less potent 5α-dihydroprogesterone (38), thus influencing the availability of ligand for the progesterone receptor (PR). This suggests that SRD5A2’s association with better survival could involve both intra-tumoral androgen and progesterone signaling pathways.

Likewise, we found higher intra-tumoral AR expression to be associated with better DSS and lower recurrence rates in patients with TP53-wild-type tumors. While the expression of androgen-metabolizing enzymes and AR in endometrial tumor tissue has been studied to some extent (39–44), our study is the first to investigate 11-oxyandrogen metabolism-related genes in this context. Recent research by Dahmani et al. has demonstrated that circulating levels of certain 11-oxyandrogens, including 11βOHA4, 11KA4, 11βOHT, 11KT, and their metabolites, 11βOH-androsterone and 11K-androsterone, decrease after tumor removal (9). The reduction of circulating 11βOHA4, a CYP11B1-mediated product, likely suggests larger changes occurring post-surgery, most probably at an adrenal gland level.

Our study has limitations. First, we studied (11-oxy)-androgen action using *in vitro* models of the epithelial cell population, which is only a small portion of the complex tumor microenvironment, however, we were able to confirm our conclusions on the TCGA UCEC cohort. Additionally, because there isn’t a commercially available control cell line derived from postmenopausal patients, we utilized a control cell line sourced from premenopausal endometrial epithelial cells. This is a limitation when comparing the steroid metabolizing capabilities of EC models established from postmenopausal patients. Altogether, our findings provide novel insights into the intricate hormonal landscape of EC and propose further exploration of 11-oxyandrogens and AR as prognostic biomarkers in EC.

In conclusion, we identified low-grade endometrial tumors of favorable molecular subtypes to have heightened potential of 11-oxyandrogen metabolism to bioactive 11KT, compared to noncancerous endometrium or high-grade, TP53-alt tumors. This implies that 11-oxyandrogens in biological fluids near endometrial tumors, such as intrauterine fluid, or even better, in the systemic circulation could hold valuable prognostic relevance in endometrioid EC. We also characterized the transcriptomic effects of potent classic and 11-oxyandrogens on EC epithelial cells. Finally, we identified high-grade tumors of NSMP molecular subtype to have abundant AR expression, and thus androgen modulating therapy might be beneficial.

## Data Availability

The datasets presented in this study can be found in online repositories. The names of the repository/repositories and accession number(s) can be found in the article/**Supplementary material**.
