# Supplementary material for: Pre-receptor regulation of 11-oxyandrogens differs between normal and cancerous endometrium and across endometrial cancer grades and molecular subtypes
Source: Front Endocrinol (Lausanne). 2024 Aug 14;15:1404804. doi: 10.3389/fendo.2024.1404804 (PMC11349532; doi:10.3389/fendo.2024.1404804)
Supplement: Supplementary file 1 [file DataSheet1.docx]

**Pre-receptor regulation of 11-oxyandrogens differs between normal and cancerous endometrium and across endometrial cancer grades and molecular subtypes**

Marija Gjorgoska^1^, Lea Sturm^1^, Tea Lanisnik Rizner^1*^

1 Institute of Biochemistry and Molecular Genetics, Faculty of Medicine, University of Ljubljana, Slovenia

*Corresponding author: [tea.lanisnik-rizner@mf.uni-lj.si](mailto:tea.lanisnik-rizner@mf.uni-lj.si)

Supplementary Material

# Supplementary Data

- 1. Supplementary Figures


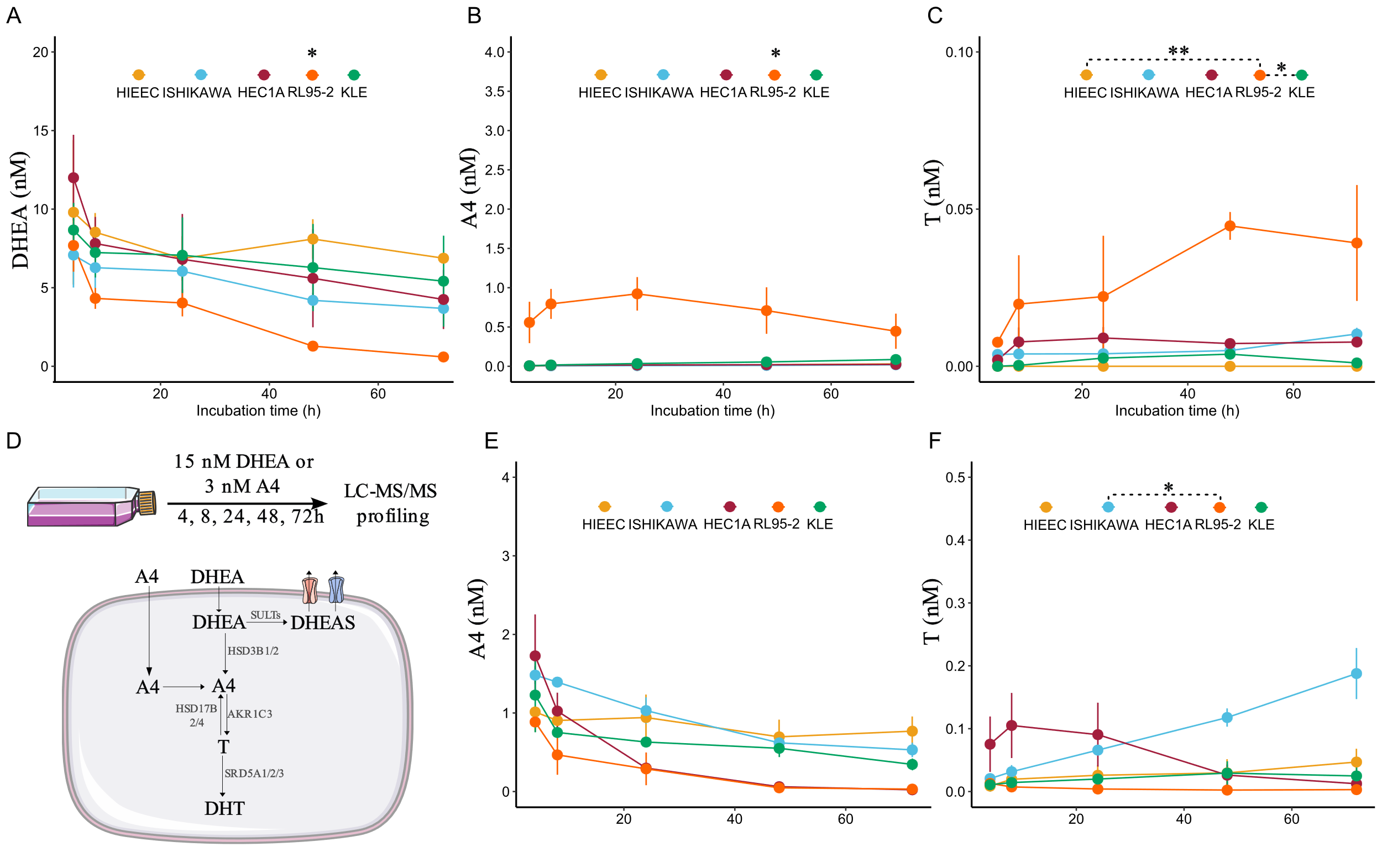


**Supplementary Figure 1:** DHEA and A4 utilization potential by control HIEEC and EC cell lines. (A-C) Profile of formed metabolites upon incubation of EC cell lines and control HIEEC cells with 15 nM DHEA (n=3, each in technical duplicate). (D) Workflow of the metabolism study. (E-F) Profile of formed metabolites upon incubation of EC cell lines and control HIEEC cells with 3 nM A4 (n=3, each in technical duplicate). Data are the mean ± SD (B-J) and raw data as dots in (B-C, J). *p<0.05, **p<0.01 by Kruskal-Wallis followed by Dunn’s post hoc test with Bonferroni correction (statistical analysis represented for the final incubation time point, 72h). A4, androstenedione; DHEA, dehydroepiandrosterone; DHEAS, DHEA-sulfate; DHT, dihydrotestosterone; T, testosterone.

**
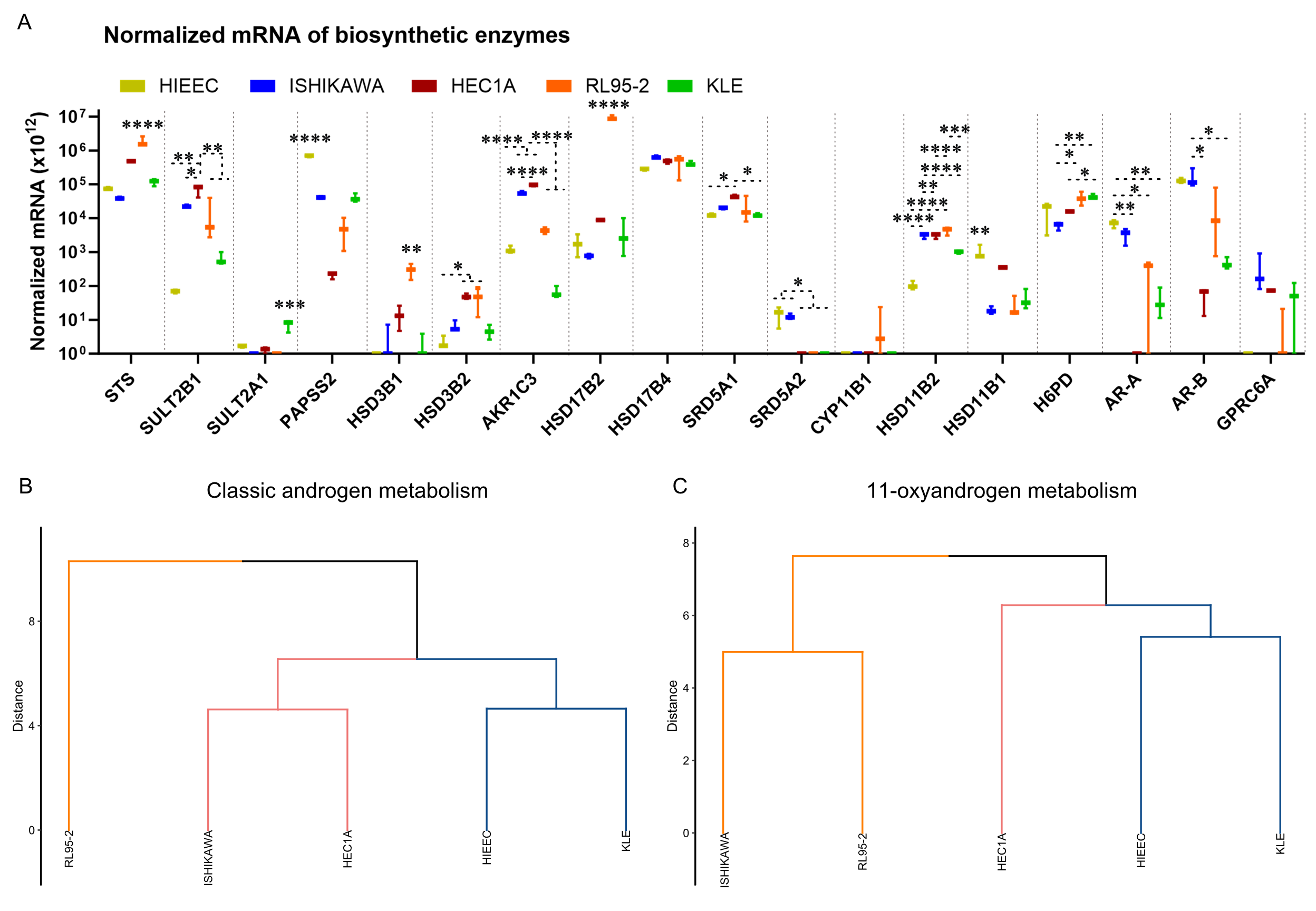
**

**Supplementary Figure 2:** Expression of key molecular players in (11-oxy)-androgen metabolism and signaling in control HIEEC and EC cell lines. (A) Gene expression of biosynthetic enzymes in the (11-oxy)-androgen metabolism and androgen receptors (n=3, each in technical triplicate). Data are mean ± SD in (A). *p<0.05, **p<0.01, ***p<0.001; ****p<0.0001 by One-Way ANOVA with Tukey’s Honestly Significant Difference (HSD) post-hoc test (A).


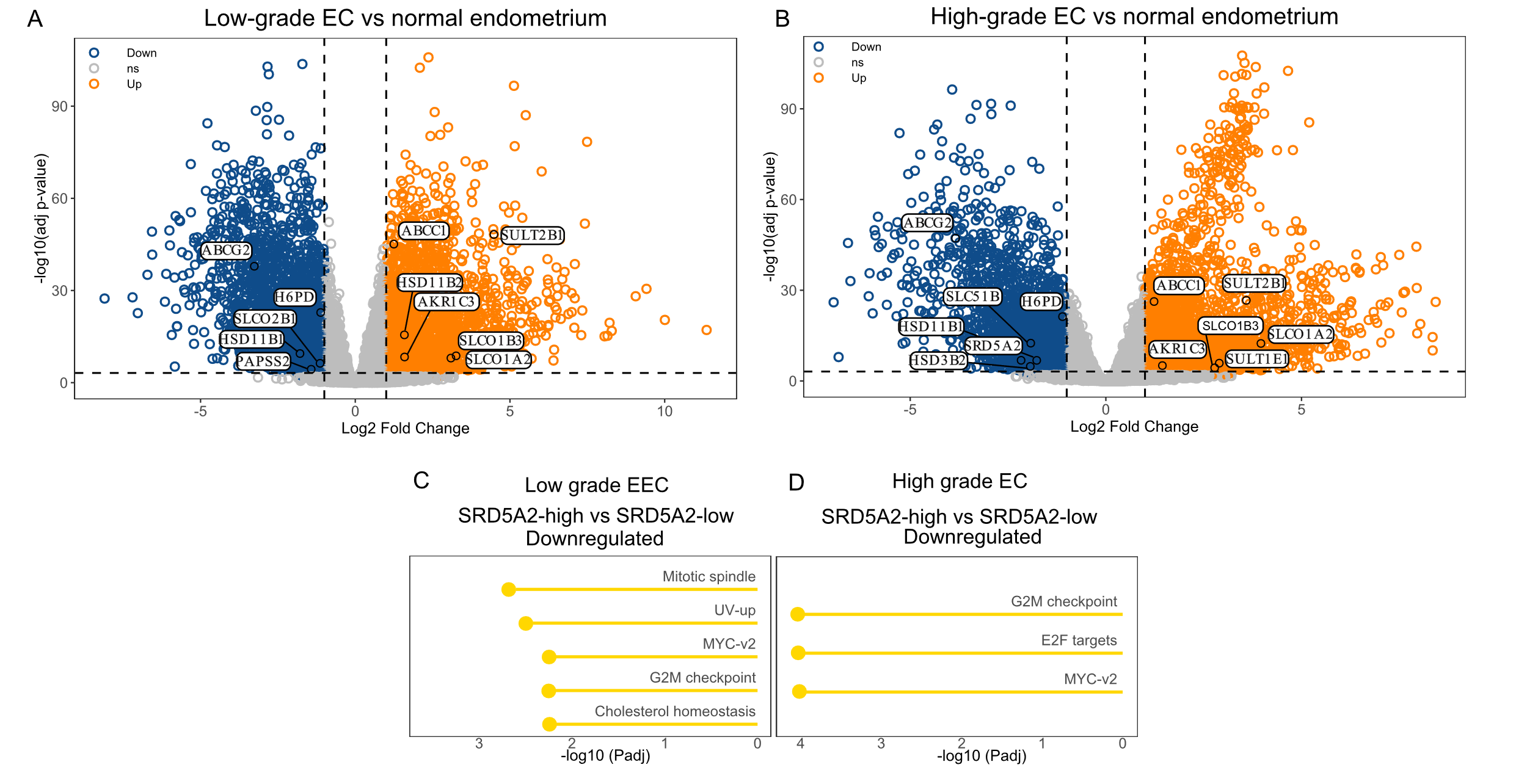


**Supplementary Figure 3:** Differential gene expression of low- and high-grade EC compared to tumor-adjacent endometrium (TCGA UCEC cohort). (A) Differential gene expression in low-grade tumors (n=215) versus tumor-adjacent endometrium (n=35) with highlighted differentially expressed genes involved in (11-oxy)-androgen metabolism. (B) Differential gene expression in high-grade tumors (n=307) and tumor-adjacent endometrium (n=as in G) with highlighted differentially expressed genes of (11-oxy)-androgen metabolism. (C) Lollipop plot showing differentially activated hallmark pathways (prioritized) in *SRD5A2*-high (n=61) vs *SRD5A2*-low (n=151) endometrioid tumors of low-grade. (D) Lollipop plot showing differentially activated hallmark pathways (prioritized) in *SRD5A2*-high (n=21) vs *SRD5A2*-low (n=183) endometrioid tumors of high-grade.
